# *Arnebia euchroma*, a Plant Species of Cold Desert in the Himalayas, Harbors Beneficial Cultivable Endophytes in Roots and Leaves

**DOI:** 10.3389/fmicb.2021.696667

**Published:** 2021-07-16

**Authors:** Rahul Jain, Priyanka Bhardwaj, Shiv Shanker Pandey, Sanjay Kumar

**Affiliations:** ^1^Biotechnology Division, Council of Scientific and Industrial Research (CSIR)-Institute of Himalayan Bioresource Technology, Palampur, India; ^2^Academy of Scientific and Innovative Research, Ghaziabad, India

**Keywords:** bacteria, yeasts, Indian Himalaya, endophytes, plant growth promotion, phytohormones

## Abstract

The endophytic mutualism of plants with microorganisms often leads to several benefits to its host including plant health and survival under extreme environments. *Arnebia euchroma* is an endangered medicinal plant that grows naturally in extreme cold and arid environments in the Himalayas. The present study was conducted to decipher the cultivable endophytic diversity associated with the leaf and root tissues of *A. euchroma*. A total of 60 bacteria and 33 fungi including nine yeasts were isolated and characterized at the molecular level. Among these, Proteobacteria was the most abundant bacterial phylum with the abundance of Gammaproteobacteria (76.67%) and genus *Pseudomonas*. Ascomycota was the most abundant phylum (72.73%) dominated by class Eurotiales (42.42%) and genus *Penicillium* among isolated fungal endophytes. Leaf tissues showed a higher richness (S_*chao*__1_) of both bacterial and fungal communities as compared to root tissues. The abilities of endophytes to display plant growth promotion (PGP) through phosphorus (P) and potassium (K) solubilization and production of ACC deaminase (ACCD), indole acetic acid (IAA), and siderophores were also investigated under *in vitro* conditions. Of all the endophytes, 21.51% produced ACCD, 89.25% solubilized P, 43.01% solubilized K, 68.82% produced IAA, and 76.34% produced siderophores. Six bacteria and one fungal endophyte displayed all the five PGP traits. The study demonstrated that *A. euchroma* is a promising source of beneficial endophytes with multiple growth-promoting traits. These endophytes can be used for improving stress tolerance in plants under nutrient-deficient and cold/arid conditions.

## Introduction

Plants are believed to coevolve with their microbial symbionts which are the integral components of a plant’s life cycle (Compant et al., 2019). The concept of plant microbiome and plant–microbe interactions has received significant attention in understanding the possible role of microbes and their genes in the survival and fitness of plants (Trivedi et al., 2020). Endophytic mutualism, involving an endophyte that colonizes internal tissues of a plant without causing any apparent symptoms to its host, is of special interest because endophytes spend all or part of their life cycle inside the plant tissues and directly influence host cells. The mutualism between endophytes and its host plant species is sustained through the production or induction of metabolites required for the growth or protection of plants against adverse environmental conditions or pathogens (Li F. et al., 2020). As evident from various reports, several metabolites of host origin and their precursors are also produced by associated endophytic symbionts (Maggini et al., 2017; Caruso et al., 2020). The role of endophytes in the primary and secondary metabolism of host plants and as a source of important secondary metabolites is also demonstrated in various plant species (Pandey et al., 2016, 2018).

Endophytes are known to colonize all plant species growing in tropical, temperate, or polar ecosystems (Acuña-Rodríguez et al., 2020). Studies on the endophytic diversity of terrestrial medicinal plants have been reported in the past few decades (Harrison and Griffin, 2020); however, diversity and ecological functions of symbionts in medicinal plants growing in the cold desert environment of the high-altitude Himalayas remain unexplored (Kotilínek et al., 2017). Soils in cold desert environments are characterized by poor availability of nutrients as well as poor mobility due to reduced microbiological activities (Margesin et al., 2009; Acharya et al., 2012). Native microbiota associated with plants in such extreme conditions plays a crucial role in nutrient uptake and fitness of the plants. Therefore, growth of high-altitude cold desert plants under adverse conditions provides an opportunity to reveal a role of endophytic colonizers in plants’ adaptation against abiotic stress and in acquisition of nutrients from desiccated cold soils (Dubey et al., 2020, 2021). Further, cultivable endophytes with specific functions and other beneficial microbiota of plants and their distribution patterns (robust colonization, consistent establishment) can be utilized to manipulate the microbiome of plants through assembling microbial synthetic communities (SynCom) (de Souza et al., 2020; Compant et al., 2021). Such studies can reveal the effects exerted by associated microbes at the community level on their host plants, especially under stress conditions for improving resiliency in crops (Harbort et al., 2020; Liu et al., 2020).

*Arnebia euchroma* (Royle) Johnston, commonly known as Pink Arnebia (family: Boraginaceae), is an endangered herb of medicinal value (Barik et al., 2018) which grows naturally on the slopes in cold desert Himalaya at an altitude ranging from 3,200 to 4,500 m above mean sea level (amsl) (Singh et al., 2012). The roots of this plant have anti-inflammatory, antimicrobial, and antipyretic properties and are traditionally used in curing eye diseases, cuts and wounds, and tooth- and earache (Gupta et al., 2013). Roots of *A. euchroma* also produce various secondary metabolites including napthoquinone pigments, meroterpenoids, and arnebinols (Wang et al., 2015). *A. euchroma* in its natural habitat experiences extreme low temperature, arid conditions, and high light intensity and grows under nutrient-limited soil with reduced water availability. The interplay of plant*–*microbe interactions in *A. euchroma* could be responsible for the uniqueness of this plant species in surviving harsh environmental conditions and in production of specific secondary metabolites. In spite of the valuable aspects of this species, its microbiota is not known. Therefore, with an aim to explore the uncharacterized microbial diversity and to decipher plant–microbe interactions in *A. euchroma*, the plants were collected from Spiti Valley in Lahaul and Spiti district of Himachal Pradesh in the western Indian Himalayas. Here we focused especially on cultivable endophytic microbial diversity due to its wider applicability for the development of microbial-based technology and to study plant–microbe interactions. To our knowledge, this is the first study to provide a detailed overview of the cultivable endophytic diversity associated with the root and leaf tissues of *A. euchroma* and their plant growth-promoting (PGP) traits. The leads of this work are being considered to evaluate the role of isolated endophytes in cold stress acclimation in plants and in accumulation of microbial assisted secondary metabolites.

## Materials and Methods

### Plant Material

*Arnebia euchroma* (Royle) Johnston plant samples growing at an altitude of ∼4,254 m amsl were collected from Langza, Spiti (N 32°16′27.93″, E 78°4′27.23″), of the Lahaul and Spiti district of Himachal Pradesh, India. The sampling was done on the third week of September, in the year 2019. For assessing culturable diversity, five randomly sampled plant specimens were transported at low temperature (4°C) and immediately processed in the lab. Root and leaf tissues were used for isolation of microbial endophytes.

### Isolation of Endophytes From Plant

For isolation of endophytes, surface sterilization of plant tissues was performed as described by Nascimento et al. (2019) with appropriate modifications. Briefly, roots and leaves were washed thoroughly with distilled water. The plant materials were surface sterilized by immersing in 70% (v/v) ethanol for 2 min, followed by washing in 1% (v/v) sodium hypochlorite for 1 min and a second washing in 70% (v/v) ethanol for 2 min. The surface-sterilized plant material was extensively rinsed with sterile distilled water three times and air-dried under sterile conditions. Distilled water used in the final wash was plated (100 μl) on agar media to validate the sterility. After surface sterilization, the plant tissues were cut into small pieces of approximately 0.5 cm in length using a sterile scalpel and kept on agar plates. Besides, 0.5 g of surface-sterilized tissue was homogenized in a sterile mortar pestle, and the homogenate was serially diluted (10-fold) in sterile saline solution (0.9% NaCl in water). One hundred microliters of all dilutions was plated on agar plates using a sterile spreader.

Nutrient agar (NA) (HiMedia, Mumbai, India) and tryptone yeast extract agar (TYEA) (HiMedia, India) were used for the isolation of bacterial endophytes. Similarly, potato dextrose agar (PDA) (HiMedia, India) and yeast extract peptone dextrose agar (YPDA) (HiMedia, India) amended with streptomycin (50 μg/ml) and chloramphenicol (15 μg/ml) were used for the isolation of fungal endophytes (Pandey et al., 2016). Agar plates were incubated at 20°C for 3–5 days in an incubator. After incubation, different morphotypes from all plates were isolated and subcultured until pure colonies were obtained. The pure endophytic isolates were uniquely coded as ARBx, ARFx and ARYx, for root and ALBx, ALFx, and ALYx for leaf-associated endophytic bacteria, fungi, and yeasts, respectively. The pure cultures were preserved as glycerol stocks at *–*80°C. For routine experiments, bacteria, fungi, and yeast cultures were grown on TYEA, PDA, and YPDA, respectively.

### Molecular Identification of Endophytes

For the identification of bacterial endophytes, genomic DNA was isolated from overnight grown bacterial culture in Luria Bertani (LB) (HiMedia, India) broth at 20°C by freeze-thaw method with appropriate modifications (two rounds of freezing at *–*80°C for 5 min and quick thawing at 95°C for 2 min followed by vortex for 1 min; Thakur et al., 2018). The cell lysate was centrifuged (10,000 × g, 5 min), and crude supernatant containing DNA was used for polymerase chain reaction (PCR). The amplification of the 16S rRNA gene was performed using universal eubacterial primer pair 27F (5′-AGAGTTTGATCCTGGCTCAG-3′) and 1492R (5′-TACCTTGTTACGACTT-3′) (Jain and Pandey, 2016). Genomic DNA was isolated from fungal isolates by the CTAB extraction method (Voigt et al., 1999). Amplification of the ITS1-5.8S-ITS2 region for fungi was performed using fungal-specific primers ITS1 (5′-TCCGTAGGTGAACCTGCGG-3′) and ITS4 (5′-GCATATCAATAAGCGGAGGA-3′) (White et al., 1990). Similarly, genomic DNA from yeast isolates was extracted by the Bust n′ Grab method (Harju et al., 2004). The D1–D2 domain of large subunit ribosomal DNA for yeast was amplified using primers NL1 (5′-GCA TATCAATAAGCGGAGGAAAAG-3′) and NL4 (5′-GGTC CGTGTTTCAAGACGG-3′) (Kurtzman and Robnett, 1997).

PCR was performed using 10 ng of genomic DNA with GoTaq^®^ Master Mix (Promega, Madison, WI, United States) in a ProFlex^TM^ thermal cycler (Applied Biosystems, Foster City, CA, United States). PCR conditions were as follows: initial denaturation at 94°C for 5 min; 30 cycles of denaturation at 94°C for 1 min, annealing at 51°C (bacteria) or 54°C (fungi/yeast) for 30 s, and extension at 72°C for 1 min, and a final extension at 72°C for 5 min. DMSO (3%) as PCR additive was used as and when required. The amplified product was checked on 1.2% agarose gel. For Sanger sequencing, the amplicons were purified with ExoSAP^TM^-IT PCR Cleanup Reagent (Applied Biosystems, United States) and used as a template for sequencing PCR using BigDye^TM^ Terminator v3.1 Cycle Sequencing Kit (Applied Biosystems, United States). The purified PCR products were sequenced on 3730xl DNA Analyzer (Applied Biosystems, United States). EzBioCloud^1^ was used to assign a taxonomic identity to 16S rRNA gene nucleotide sequences of bacteria. For fungi and yeasts, the nucleotide sequences were identified using the BLASTn program at NCBI^2^.

### Molecular Phylogeny and Alpha Diversity

For the phylogenetic relationship, bacterial and fungal nucleotide sequences were aligned separately using the MUSCLE algorithm in MEGA X software (Kumar et al., 2018). Maximum likelihood phylogenetic analysis was performed using RAxML v8 (Stamatakis, 2014) in Geneious Prime 2021 software. The confidence at each node of the phylogenetic tree was evaluated by bootstrap analysis with 1,000 replicates. Interactive Tree of Life (iTOL) v5, an online tree explorer^3^, was used to display and annotate phylogenetic trees (Letunic and Bork, 2021).

Alpha diversity of isolated endophytic bacteria and fungi associated with plant tissues and the complete plant was calculated at the genus level to assess the observed richness (number of taxa, S) and evenness (abundance of taxa) of microbial communities. Shannon diversity index (H), Simpson’s index (D), Simpson’s diversity index (1*–*D), evenness (e^H/S), and predicted species richness (S_*Chao*__1_) were determined individually for bacteria and fungi in leaf and root samples as well as for the whole plant. All the diversity analysis was performed using the PAST software package v4.03 (Hammer et al., 2001).

### PGP Traits of Endophytes

#### 1-Aminocyclopropane-1-Carboxylate Deaminase (ACCD) Production

For estimating ACCD production by endophytes, all the isolates were inoculated in 10 ml synthetic media (composition per liter: glucose, 15 g; MgSO_4_⋅7H_2_O, 0.2 g; K_2_HPO_4_, 0.6 g; KCl, 0.15 g; NH_4_NO_3_, 1 g; 1 ml of trace solution containing per liter: FeSO_4_⋅7H_2_O, 0.005 g; MnSO_4_⋅H_2_O, 0.006 g; ZnSO_4_⋅H_2_O, 0.004 g; CoCl_2_, 0.002 g) (Yedidia et al., 1999) in a 50-ml Falcon tube. One percent (100 μl) of freshly grown bacteria (OD_600_ = 0.5) and a 5-mm disk of 7-day grown fungi, separately, were used as inoculum. Following inoculation, cultures were incubated for 24 h in the case of bacteria, and for 48 h in the case of fungi at 20°C under shaking conditions (200 rpm). After incubation, the culture broth was centrifuged (6,000 × g, 5 min), the supernatant was discarded, and the pellet was resuspended in 5 ml of sterile synthetic media containing 3 mM ACC but without ammonium nitrate (NH_4_NO_3_). The microbial suspensions were reincubated for 24 h in the case of bacteria and 48 h in the case of fungi at the same conditions. Following incubations, culture broths were centrifuged (10,000 × g, 10 min), the supernatant was discarded, and biomass was resuspended in 2.5 ml of Tris–Cl (0.1 M, pH 8.5) buffer.

ACCD activity was measured by the method as described by Nascimento et al. (2019) with appropriate modifications. Briefly, bacterial/fungal biomass in Tris-Cl buffer was homogenized using a bead beater (25 s × 2 cycles) with a 5-min incubation on ice between two cycles. Toluene (25 μl) was added to the cell lysate and vortexed vigorously for 30 s. ACC (20 μl of 0.5 M solution in water) was added to 200 μl of lysate, and after an incubation period of 15 min at 30°C, 1 ml of 0.56 N HCl was added. The mixture was centrifuged (10,000 × g, 10 min), and 1 ml of supernatant was mixed with 800 μl of 0.56 N HCl and 300 μl of 2,4-dinitrophenylhydrazine (DNPH) solution (0.2 g DNPH dissolved in 100 ml of 2 N HCl). The mixture was incubated for 30 min at 30°C, and 2 ml of 2 N NaOH was added. The absorbance at 540 nm (*A*_540_) was measured in a 96-well plate using a Synergy H1 microplate reader (BioTek Instruments, Winooski, VT, United States). ACCD activity was calculated by measuring the amount of α-ketobutyrate released by the deamination of ACC using a standard curve made using α-ketobutyrate. ACCD activity was expressed as μmol α-ketobutyrate/h/mg protein. Protein concentrations in the cell lysates were determined using the Bradford method (Bradford, 1976). Three independent replicates were used for activity measurements.

#### Phosphate (P) Solubilization and Determination of pH

Pikovskaya’s agar (HiMedia, India) (pH 7.0) containing tri-calcium phosphate (TCP) was used for screening of P-solubilization by bacterial and fungal endophytes. A loopful of bacterial inoculum or a 5-mm fungal disc was inoculated on Pikovskaya’s agar and incubated at 20°C for 5 days. After incubation, a clear zone of solubilization around the bacterial/fungal colonies indicated P-solubilization. The colony diameter (CD) and zone diameter (ZD) were measured to calculate the solubilization index (SI = ZD/CD). The positive isolates for P-solubilization were further investigated for quantification of soluble phosphorus in National Botanical Research Institute’s Phosphate growth medium (NBRIP) broth (pH 7.0) containing 0.5% (w/v) TCP (Nautiyal, 1999). NBRIP broth (50 ml) in a 250-ml Erlenmeyer flask was inoculated with 500 μl overnight grown bacteria (OD_600_ = 0.5) or a 5-day-grown fungal disc (5 mm) and incubated at 20°C at 200 rpm for 5 days. Following incubation, the soluble P in the cell-free culture supernatant was quantified by the method described by Adhikari et al. (2021). *A*_882_ was measured in a 96-well plate using a Synergy H1 microplate reader. Soluble P (μg/ml) in broth was estimated using a standard curve of KH_2_PO_4_. The pH of the culture supernatants was also measured using a pH meter.

#### Indole Acetic Acid (IAA) Production and Potassium (K) Solubilization

For estimation of IAA production, 200 μl of overnight grown bacteria (OD_600_ = 0.5) and a 5-mm fungal disc was inoculated in 20 ml LB broth (HiMedia, India) and potato dextrose broth (PDB) (HiMedia, India), respectively, supplemented with 0.5 mg/ml of L-tryptophan. The growth was allowed at 20°C for 2 days in the case of bacteria and 5 days in the case of fungi at 200 rpm. Un-inoculated broth containing L-tryptophan served as a control. After incubation, the production of IAA by the microbial endophytes was quantified by mixing 100 μl of culture supernatant with 100 μl of freshly prepared Salkowski reagent [2 ml of 0.5 M FeCl_3_ in 98 ml of 35% (v/v) HClO_4_] (Ye et al., 2019). After 20 min of incubation in the dark at room temperature, *A*_530_ was measured in a 96-well plate using a Synergy H1 microplate reader. A standard curve of IAA was prepared for quantifying IAA production (μg ml^–1^) in the samples. All the experiments were performed in triplicates.

Microbial screening for K-solubilization was performed in plate assays using Aleksandrow agar (HiMedia, India). All isolates were inoculated as described previously and incubated at 20°C for 5 days. The zone of solubilization around the microbial colonies was recorded following incubation. Solubilization indices were calculated as indicated above.

#### Siderophore Production

The solid and liquid versions of CAS assays (Jain and Pandey, 2016) were employed for measuring siderophore production by the endophytic isolates. All the glasswares used for the preparation of siderophore production media were soaked overnight in 10% (v/v) HCl and rinsed five times with Milli-Q water. Casamino acid used in the media was defarrated by extracting with 3% 8-hydroxyquinoline in chloroform and filter sterilized (Louden et al., 2011). For plate-based assays, freshly grown microbial inoculum was spotted on CAS agar (Schwyn and Neilands, 1987) and incubated for 5 days at 20°C in the dark. The orange zone around the colonies showed siderophore production by the isolates. The siderophore production index was calculated as explained above.

For the liquid version of CAS assay, iron-free Czapek-Dox broth containing 3% sucrose, 0.2% NaNO_3_, 0.1% K_2_HPO_4_, 0.05% MgSO_4_⋅7H_2_O, and 0.05% KCl (pH 7.3) (Larcher et al., 2013) was used for siderophore production by fungi. Similarly, iron-free M9 minimal broth (pH 7.0) (Sinha et al., 2019) was used for quantifying siderophore production by bacteria. The endophytic isolates were inoculated (100 μl bacterial suspension and a 5 mm fungal disc) in 10 ml of respective broth in 30-ml glass vials and incubated at 20°C for 5 days at 200 rpm. After incubation, culture supernatants were collected following centrifugation (8,000 × g, 10 min). One hundred microliters of supernatant was mixed with 100 μl of CAS dye solution, and the mixture was incubated in the dark for 15 min. *A*_630_ was measured using the Synergy H1 microplate reader against control containing 100 μl of un-inoculated broth mixed with 100 μl of CAS dye solution. Quenching of iron in CAS dye solution by the siderophores in the samples results in discoloration of the resulting mixture in comparison to the control. Percent siderophore units (SU) were calculated by the formula %SU = (*A*r−*A*s)/*A*r × 100 (where *A*r is *A*_630_ of control and *A*s is *A*_630_ of samples). %SU < 10 was considered as negative. All the measurements were performed in triplicates.

### Statistical Analysis

Analysis of variance (ANOVA) with *post hoc* Duncan’s multiple-range test (DMRT) and Pearson correlation coefficient (r) were calculated using SPSS v20. Venn diagrams were drawn using the jvenn online tool^4^. All the data are presented as mean ± standard deviation of three replicates.

## Results

### Endophytic Diversity

A total of 60 bacterial endophytes encompassing 21 from leaf and 39 from root tissues; 24 endophytic fungi encompassing 15 from leaf and 9 from root tissue; and 9 yeast endophytes encompassing 4 from leaf and 5 from root tissues of *A. euchroma* were isolated. All these endophytic isolates were identified by sequencing either 16S rRNA gene (bacteria), ITS1-5.8S-ITS2 region (fungi), or D1/D2 domain of the large subunit of ribosomal DNA (yeasts). Nucleotide sequences of all endophytic isolates are deposited in the GenBank database with accession numbers MW665325*–*MW665384 for bacteria; MW391724*–*MW391747 for fungi; and MW391800*–*MW391808 for yeast isolates.

All the bacterial endophytes were identified to species level with more than 99% similarity using the EzTaxon/NCBI database except ALB20, ARB6, and ARB37, which were identified to genus level as *Pseudomonas* spp. (Supplementary Table 1). Similarly, except for ARF7 (*Cladosporium* sp.); ARF9, ALF8, and ALF9 (*Penicillium* spp.); ALF14 (*Xylaria* sp.); ALF12 (*Carrena* sp.); and ALF15 (*Periconia* sp.), all fungal endophytes were assigned species-level identity (Supplementary Table 2).

**FIGURE 1:**
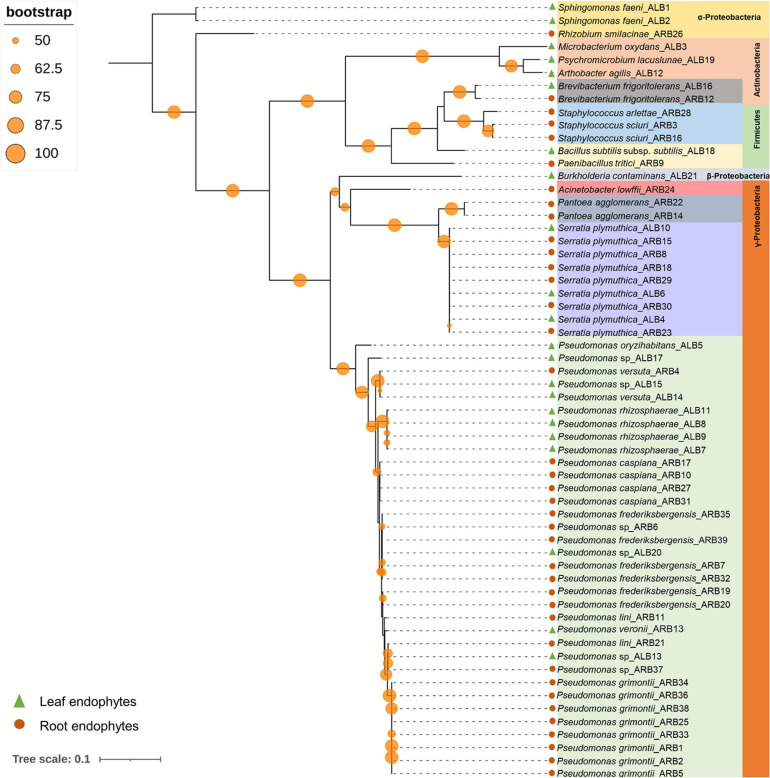
Phylogenetic relation among bacterial endophytes isolated from *A. euchroma* leaf and root tissues. A total of 60 bacteria were isolated and analyzed for reconstruction of phylogeny using the Maximum Likelihood method with bootstrap replication of 1,000. A reoccurrence frequency of ≥50% is indicated on the node by a circle. A solid circle and triangle before taxonomic designation indicate root and leaf endophyte, respectively.

**FIGURE 2:**
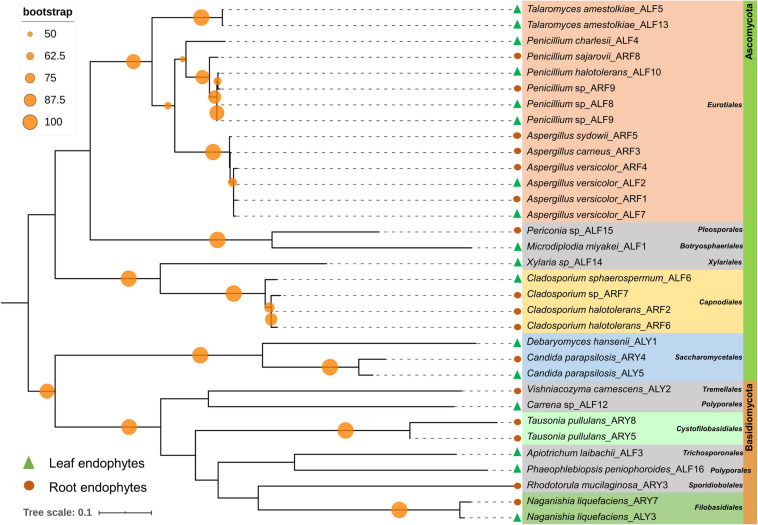
Phylogenetic relation among fungal endophytes isolated from *A. euchroma* leaf and root tissues. A total of 33 fungi were isolated and analyzed for reconstruction of phylogeny using the Maximum Likelihood method with bootstrap replication of 1,000. A reoccurrence frequency of ≥ 50% is indicated on the node by a circle. A solid circle and triangle before taxonomic designation indicate root and leaf endophyte, respectively.

Among 60 bacterial endophytes, 83.34% bacterial endophytes belonged to class Proteobacteria, which were further classified into Gammaproteobacteria (76.67%), Alphaproteobacteria (5%), and Betaproteobacteria (1.67%). The remaining 16.66% bacterial endophytes belonged to Firmicutes and Actinobacteria (8.33% each). During phylogenetic analysis, all closely related bacteria formed well-supported clades (Figure 1). Similarly, out of 33 fungal endophytes, 72.73% fungi were classified under Ascomycota phylum, which were further grouped in Eurotiales (42.42%), Capnodiales (12.12%), Saccharomycetales (9.09%), and 3.03% each of Xyriales, Pleosporales, and Botryosphaeriales. The remaining 27.27% fungal endophytes were classified under Basidiomycota phylum, which was further grouped in 6.06% each of Cystofilobasdiales, Filobasidiales, and Polypropales and 3.03% each of Tremallales, Trichosporonales, and Sporidiobolales. In phylogenetic tree reconstruction, all fungal genera of Ascomycota and Basidiomycota phylum formed well-separated clades (Figure 2).

### Tissue Specificity of Endophytes

*Pseudomonas* followed by *Serratia* were most prominently isolated from roots as well as leaf tissues of plant (Supplementary Figure 1A). *Pseudomonas* accounted for 61.54 and 47.62% of all isolated bacterial endophytes in root and leaf tissues, respectively. Other bacteria that colonized the plant tissues included *Staphylococcus*, *Pantoea*, *Sphingomonas*, *Brevibacterium*, *Paenibacillus*, *Bacillus*, *Acinetobacter*, *Burkholderia*, *Microbacterium*, *Pschromicrobium*, *Arthrobacter*, and *Rhizobium*. Similarly, leaf tissues showed a dominance of *Penicillium* while root tissues were dominantly colonized with *Aspergillus* (Supplementary Figure 1B). The total number of genera isolated from leaf tissues was higher (*S* = 14) as compared to the roots (*S* = 7).

**FIGURE 3:**
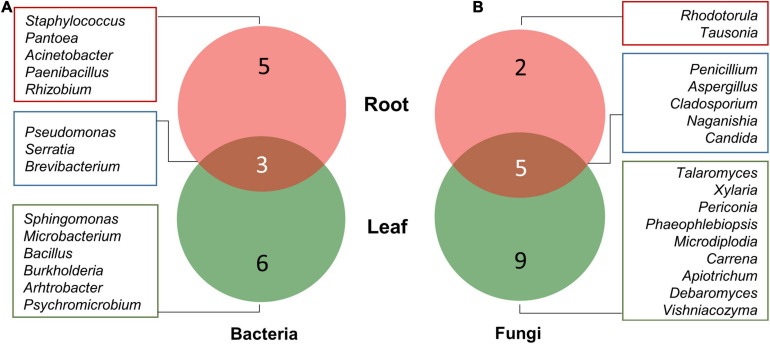
Venn diagram illustrating the number of unique and overlapping bacterial **(A)** and fungal **(B)** endophytes isolated from leaf and root tissues of *A. euchroma*.

Among bacteria, five genera, namely, *Rhizobium*, *Paenibacillus*, *Staphylococcus*, *Pantoea*, and *Acinetobacter*, were isolated from root tissues only, while six genera including *Sphingomonas*, *Arthrobacter*, *Burkholderia*, *Bacillus*, *Microbacterium*, and *Psychromicrobium* were isolated only from leaf tissues. *Pseudomonas*, *Serratia*, and *Brevibacterium* were isolated from both roots as well as leaf tissues (Figure 3A). Similarly, nine fungal genera, namely, *Talaromyces*, *Xylaria*, *Periconia*, *Phaeophlebiopsis*, *Microdiplodia*, *Carrena*, *Apiotrichum*, *Debaryomyces*, and *Vishniacozyma* were found uniquely associated with only leaf tissues, while *Rhodotorula* and *Tausonia* were only isolated from root tissues. Fungal genera including *Penicillium*, *Aspergillus*, *Cladosporium*, *Naganishia*, and *Candida* were isolated from both roots and leaf tissues (Figure 3B).

### Alpha Diversity

Shannon’s diversity index (H), Simpson’s indices (D and 1*–*D), Evenness (e^H/S), and species richness (S_*chao*__1_) were calculated to assess the diversity, evenness, and richness of cultivable endophytes isolated from the plant tissues of *A. euchroma* (Table 1 and Supplementary Figure 2). A higher H (1.73), 1*–*D (0.73), and e^H/S (0.62) value for bacterial communities in leaf tissue as compared to roots (H = 1.31; 1*–*D = 0.59; e^H/S = 0.46) indicated a higher diversity and even distribution of bacteria in leaf tissues. A similar pattern was also observed for fungal communities with higher diversity and even distribution in leaves. In addition, the total diversity of isolated endophytes in leaf tissue was also higher (H = 2.79; 1*–*D = 0.90) as compared to root tissue (H = 2.02; 1*–*D = 0.76) and their distribution was more even. Furthermore, a high richness (S_*chao*__1_) of bacteria (16.50) and fungi (32.33) in leaf tissues as compared to bacteria and fungi in roots (S_*chao*__1_ = 11.00 and 8.00, respectively) was observed. The overall predicted microbial richness was higher in leaf tissues (S_*chao*__1_ = 57.00) than in root tissues (S_*chao*__1_ = 20.25). However, these diversity measures are the description of results obtained in the present study and could be biased by the sampling as well as isolation methods.

**TABLE 1:** Alpha diversity indices of microbial endophytes in different tissues of *A. euchroma*.

Diversity indices	Leaf bacteria	Root bacteria	Leaf fungi	Root fungi	Bacteria	Fungi	Leaf	Root	Whole plant
*Taxa* (S)	9	8	14	7	14	16	23	15	30
*Simpson’s index* (D)	0.27	0.41	0.10	0.18	0.35	0.10	0.10	0.24	0.16
*Simpson’s diversity index* (1-D)	0.73	0.59	0.90	0.82	0.65	0.90	0.90	0.76	0.84
*Shannon’s diversity index* (H)	1.73	1.31	2.51	1.81	1.64	2.51	2.79	2.02	2.60
*Evenness* (e^H/S)	0.62	0.46	0.88	0.87	0.37	0.77	0.71	0.50	0.45
*Species richness* (S_*Chao*__1_)	16.50	11.00	32.33	8.00	21.00	23.20	57.00	20.25	47.00

### PGP Traits of Endophytes

#### ACC Deaminase Production

**FIGURE 4:**
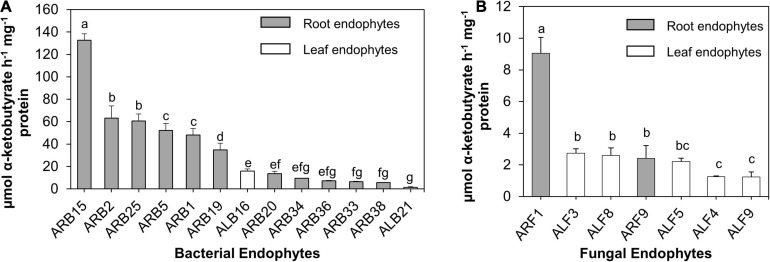
ACC deaminase (ACCD) activity (μmol α-ketobutyrate/h/mg protein) of endophytes isolated from the leaf and root tissues of *A. euchroma*. Total protein after cell lysis was measured using Bradford assay. **(A)** ACCD activity of bacterial endophytes and **(B)** fungal endophytes. Bars with different alphabets indicate a significant difference (*p* < 0.05) as calculated using Duncan’s multiple-range test. Error bars indicate standard deviation (*n* = 3).

Among all bacterial endophytes, only 13 bacterial isolates that included 11 root bacterial endophytes and 2 leaf-associated bacteria displayed ACC deaminase activity (Figure 4A). Among all, isolate ARB15 (*Serratia plymuthica*) showed the maximum ACCD activity (132.69 μmol α-ketobutyrate/h/mg protein) followed by ARB2 (63.25 μmol α-ketobutyrate/h/mg protein) and ARB25 (60.58 μmol α-ketobutyrate/h/mg protein) (both identified as *Pseudomonas grimontii*). Leaf endophytes, i.e., ALB16 (*Brevibacterium frigoritolerans*) and ALB21 (*Burkholderia contaminans*), displayed ACCD activity of 15.88 and 1.39 μmol α-ketobutyrate/h/mg protein.

Similarly, for fungal endophytes, only seven isolates exhibited ACCD activity (Figure 4B) that included two root endophytes (ARF1 and ARF9) and five leaf endophytes (ALF3, ALF4, ALF5, ALF8, and ALF9). ARF1 (*Aspergillus versicolor*) displayed the highest activity of 9.04 μmol α-ketobutyrate/h/mg protein, which was significantly higher (*p* < 0.05) than that of other fungal endophytes.

#### P-Solubilization and Correlation With pH of the Culture Supernatants

**FIGURE 5:**
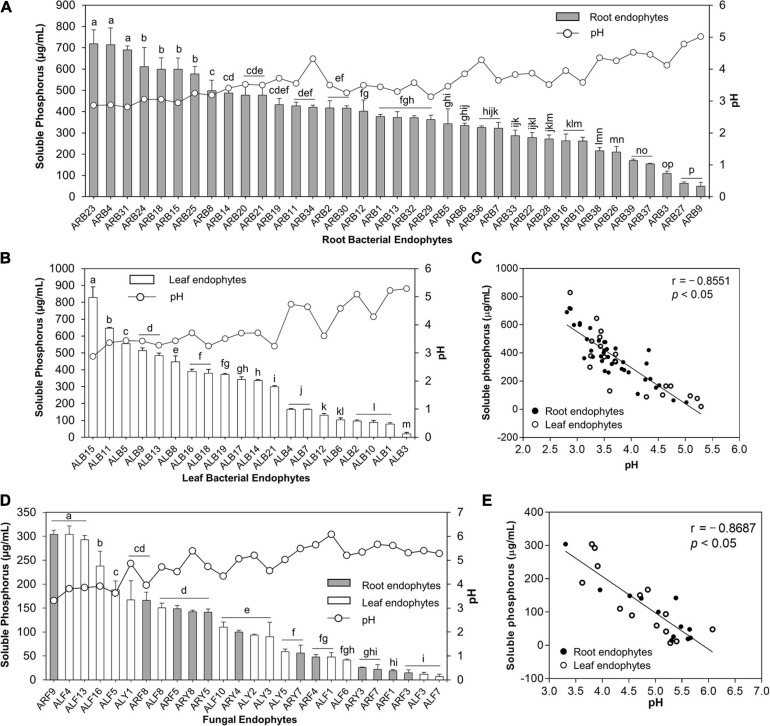
Phosphate solubilization by bacterial and fungal endophytes isolated from leaf and root tissues of *A. euchroma*. Soluble P (μg/ml) in the culture supernatants was quantified, and pH of supernatants was measured. Phosphate solubilization by root **(A)** and leaf **(B)** associated bacterial endophytes and corresponding pH of the supernatants. **(C)** Correlation between pH and P solubilized by bacterial endophytes. **(D)** Phosphate solubilization of fungal endophytes and corresponding pH of the supernatants. **(E)** Correlation between pH and P solubilized by fungal endophytes. Bars with different alphabets indicate a significant difference (*p* < 0.05) as calculated using Duncan’s multiple-range test. Error bars represent standard deviation (*n* = 3).

A remarkable feature of isolated endophytes in this study was their potential to solubilize TCP at 20°C (Supplementary Tables 3, 4 and Figure 5). Out of 39 root-associated bacteria, 37 bacteria (94.87%) solubilized P in the range of 49.57 to 718.55 μg/ml (Supplementary Figure 3A). ARB23 (*Serratia plymuthica*; 718.55 μg/ml), ARB4 (*Pseudomonas versuta*; 713.91 μg/ml), and ARB31 (*P. caspiana*; 689.86 μg/ml) solubilized maximum P with significant difference (*p* < 0.05) than other root-associated bacterial endophytes. The lowest pH of 2.81 was recorded for the culture supernatants of isolate ARB31, while the highest pH of 5.02 was recorded for ARB9 (*Paenibacillus tritici*), which also solubilized minimum P (Figure 5A). Among 21 leaf endophytes, 20 isolates (95.24%) possessed P-solubilization efficiency (Figure 5B). ALB15 (*Pseudomonas* sp.) solubilized significantly higher (*p* < 0.05) P, i.e., 828.99 μg/ml, as compared to all other endophytes and exhibited the lowest reduction in the pH of culture broth to 2.87.

Similarly, out of 33 fungal endophytes, 26 isolates (78.78%) including 12 root and 14 leaf-associated endophytes solubilized TCP in the broth (Figure 5D). The overall efficiency of fungal endophytes to solubilize tricalcium phosphate was lower than the bacterial endophytes that ranged from 6.96 to 304.06 μg/ml (Supplementary Figure 3B). The highest soluble P, i.e., 304.06 μg/ml, in the broth was recorded for isolate ARF9 (*Penicillium* sp.) which also caused the lowest reduction in the pH of culture broth to 3.31 as compared to pH 7 for control. Isolate ALF4 (*Penicillium charlesii*) and ALF13 (*Talaromyeces amestolkiae*) also solubilized the highest P with no significant difference than ARF9; however, the pH of the culture broth was recorded as 3.80 and 3.86, respectively.

A significant and strong negative correlation i.e., *r* = *–*0.8551 and *r* = *–*0.8687 (*p* < 0.05), between the pH of the culture supernatants and P-solubilization by bacterial (Figure 5C) and fungal endophytes (Figure 5E), respectively, was observed. This reduction in the pH of the culture supernatant suggested the production of low molecular weight organic acids by the endophytic isolates that lead to P-solubilization.

#### IAA Production and K-Solubilization

**FIGURE 6:**
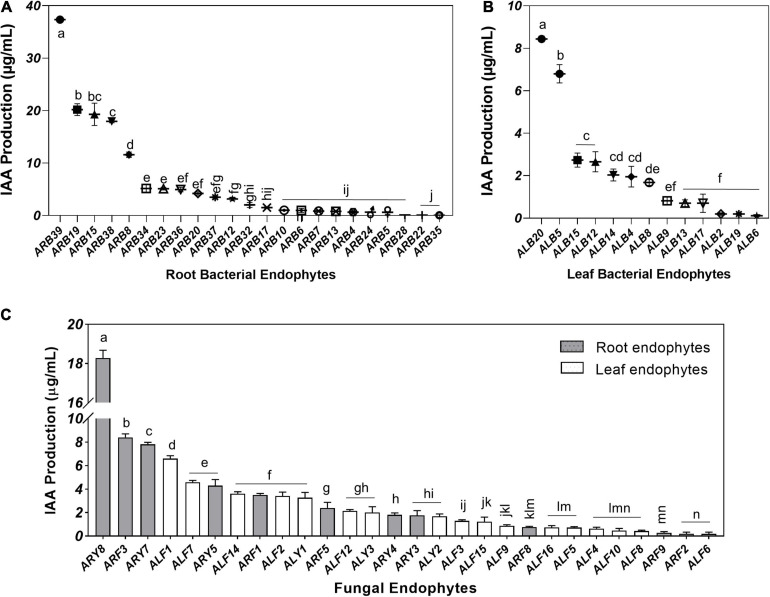
Indole acetic acid (IAA) production by microbial endophytes of *A. euchr*oma quantified using Salkowski reagent. IAA production by root **(A)** and leaf **(B)** associated bacterial endophytes. **(C)** IAA production by root (gray) and leaf (white) associated fungal endophytes. Bars with different alphabets indicate a significant difference (*p* < 0.05) as calculated using Duncan’s multiple-range test. Error bars represent standard deviation (*n* = 3).

Root-associated endophytes were the more efficient producer of IAA as compared to leaf endophytes (Supplementary Figures 3C,D). Among 39 root-associated bacterial endophytes, 23 isolates (58.97%) displayed IAA production in the media supplemented with 0.5 mg/ml tryptophan in the range of 0.05–37.36 μg/ml (Figure 6A). Similarly, 13 out of 21 (61.90%) leaf-associated bacterial endophytes produced IAA ranging from 0.12 to 8.44 μg/ml (Figure 6B). Bacterial isolate ARB39 (*Pseudomonas frideriksbergensis*) and its phylogenetic neighbor ALB20 (*Pseudomonas* sp.) produced maximum IAA, i.e., 37.36 and 8.44 μg/ml, among root and leaf-associated bacterial endophytes, respectively. On the other side, 11 out of 14 (78.57%) root-associated fungi and 17 out of 19 (89.47%) leaf-associated fungi produced IAA (Figure 6C). Root-associated yeast endophyte ARY8 was the most efficient producer of IAA (18.28 μg/ml) while ALF1 among leaf endophytes produced the highest amount of IAA, i.e., 6.60 μg/ml.

K-solubilization by endophytic isolates was tested using plate-based assays. Thirty-six bacterial endophytes out of 60 (60%), that majorly included root-associated bacteria (50%) and 10% leaf-associated bacteria, showed K-solubilization efficiency (Supplementary Table 3). On the other side, only four fungal isolates out of total 33 endophytes, namely, ARF8 (*Penicillium sajarovii*), ARF9 (*Penicillium* sp.), ALF13 (*Talaromyces amestolkiae*), and ARY7 (*Naganishia liquefaciens*) solubilized K in the medium (Supplementary Table 4).

#### Siderophore Production

**FIGURE 7:**
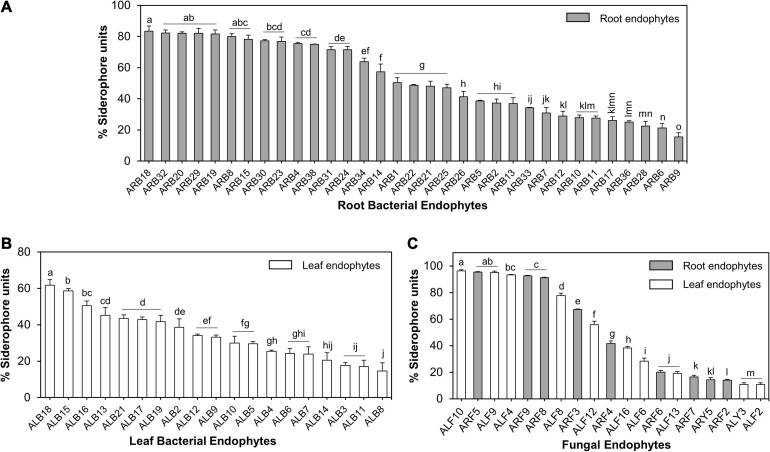
Siderophore production by microbial endophytes of *A. euchr*oma estimated using liquid CAS assay. Siderophore production by root **(A)** and leaf **(B)** associated bacterial endophytes. **(C)** Siderophore production by root (gray bars) and leaf (white bars) associated fungal endophytes. Different alphabets indicate a significant difference (*p* ≤ 0.05) as calculated using Duncan’s multiple-range test. Error bars represent standard deviation (*n* = 3).

Of the 39 root-associated bacterial endophytes, 36 endophytic isolates showed production of siderophores as estimated using solid CAS assay (Supplementary Table 3), while 33 were confirmed as true siderophore producers through liquid CAS assay as the remaining isolates produced <10% SU. The %SU quantified by these isolates ranged between 15.57 and 83.38% (Supplementary Figure 3E). Of these 33 endophytes, 17 isolates produced SU ≥50%. Isolate ARB18 (*Serratia plymuthica*) was the highest producer of siderophores with a %SU of 83.38% (Figure 7A). Similarly, out of 21 leaf-associated bacterial endophytes, 19 isolates showed siderophore production in the range of 14.64–61.67% SU (Supplementary Figure 3E). Isolate ALB18 (*Bacillus subtilis* subsp. *subtilis*) was the most significant producer of siderophore as compared to other leaf-associated bacterial endophytes (Figure 7B).

Similarly, of all fungal endophytes, 19 isolates produced siderophores in liquid CAS assay that included 9 root-associated and 10 leaf-associated fungi (Supplementary Figure 3F). *Aspergillus sydowii* ARF5 and *Penicillium halotolerans* ALF10 were the maximum producers of siderophore, i.e., 96.41 and 95.39% SU. Nine out of 19 fungal isolates produced > 50% SU (Figure 7C).

## Discussion

Microbial symbionts of plants growing in cold and extreme environments, including one in the present study, are interesting candidates with a promising role in cold stress alleviation in plants (Zhang et al., 2013; Acuña-Rodríguez et al., 2020). The diversity of endophytes in plants growing in these specific environments and their contributions to plant adaptability are not much explored (Cui et al., 2015). Therefore, the present study was aimed to investigate the diverse cultivable endophytic microbiota associated with *A. euchroma* and study their functional aspects in plant growth promotion. The ultimate goal of this work is to identify possible mechanisms contributing to the amelioration of cold stress tolerance in plants.

### Microbial Communities in *A. euchroma*

The occurrence of 83.34% Proteobacteria mostly represented by Gammaproteobacteria (76.67%) was observed in this study. *Pseudomonas* was the most commonly isolated bacterial genera from both root and leaf tissues of *A. euchroma* (Supplementary Figure 1). It has been observed that plants majorly recruit Proteobacteria especially Gammaproteobacteria in their plant tissues (Bafana, 2013; Xu et al., 2019). For example, Khan Chowdhury et al. (2017) reported the abundance of Proteobacteria accounting for ∼63% of total OTUs with *Pseudomonas* as the most abundant genera in mountain-cultivated ginseng (*Panax ginseng* Meyer) using the culture-dependent approach. Similarly, Webster et al. (2020) also concluded the abundance of endophytic bacterial genera belonging to Gammaproteobacteria in medicinal plants of Western Ghats, India. Similar observations on bacterial diversity of endophytes have also been reported through metagenomics study (Zhang et al., 2019). Next to *Pseudomonas*, *Serratia* was another commonly isolated endophyte in this study. *Serratia* as the endophytic successor in various medicinal plants and crops is reported in various studies (Asaf et al., 2017; Eckelmann et al., 2018).

The occurrence of 21 fungal taxa in *A. euchroma* tissues indicates high diversity of cultivable fungi, which was dominated by phylum Ascomycota (72.73%). These isolated fungi under the Ascomycota phylum mostly represented class Eurotiales and genera *Penicillium*, *Aspergillus*, and *Talaromyces*. High occurrence of Ascomycetes fungal endophytes is reported earlier in different medicinal plants. For example, Tan et al. (2018) reported 93% occurrence of Ascomycota in *Dysosma versipellis*. An et al. (2020) reported endophytic fungal diversity of *Chloranthus japonicus*, where 97.89% of endophytes belonged to the Ascomycota phylum. In contrast to the dominance of *Colletotrichum* spp. in these medicinal plants (Tan et al., 2018; An et al., 2020), *Penicillium* followed by *Aspergillus* were the most dominating fungal taxa in the current study. In another study on fungal endophytes isolated from *Rhodiola* spp. (Cui et al., 2015), phylum Ascomycota represented 88.89% of all fungal endophytes followed by Basidiomycota (2.78%).

An interesting observation in this study was the colonization of plant tissues with yeast microbial communities that belonged to both Ascomycota and Basidiomycota phylum (Supplementary Table 2). Limited studies have reported cultivable yeast endophytes in plants, which suggested their role in biocontrol of pathogens and promotion of plant growth (Luna, 2017). A basidiomycetes-pigmented yeast *Rhodotorula mucilaginosa* and Ascomycetes yeasts *Debaryomyces hansenii* and *Candida* spp. were reported as endophytes from different plant parts of Brazilian apple (*Malus domestica*) (Camatti-Sartori et al., 2005). Close relatives of *Vishniacozyma carnescens*, i.e., *V. alagoana* and *V. victoriae*, were recently reported as endophytes of bromeliads (Félix et al., 2020) and maple tree (Wemheuer et al., 2019), respectively. In addition, some of the yeasts isolated in this study (*Tausonia pullulans* and *Naganishia liquefaciens*) were not reported earlier as endophytic colonizers in plants; however, their existence in soils and extreme habitats has been reported (Han et al., 2020; Li A. H. et al., 2020).

### Diversity Assessment of Endophytes

The overall cultivable endophytes in *A. euchroma* were represented by 30 taxa with a Simpson’s and Shannon’s diversity index (1*–*D) of 0.84 and 2.60, respectively. The predicted species richness (S_*chao*__1_) of the whole plant was estimated at 47 while it was 57 and 20.25 individually for leaf and root tissues, respectively (Table 1). The diversity data suggested a highly diverse microbial community inhabiting *A. euchroma* plant with higher species richness in leaves as compared to roots. This was true for both bacterial and fungal endophytic microbiota (Table 1). These results corroborate with the study of Purushotham et al. (2020) where the richness of endophytic Alpha, Beta, and Gammaproteobacteria was higher in leaves in comparison to stem and roots of primitive New Zealand medicinal plant *Pseudowintera colorata.* Fernandes et al. (2015) and Sharma et al. (2018) also observed higher diversity and greater species richness for fungal endophytes in leaf as compared to other plant tissues in *Glycine max* and *Berberis aristata* DC. Plant leaves under stressed environments can exhibit low foliar nutrients or high levels of toxic compounds. Both of these responses can account for endophyte microbial richness by limiting the growth of otherwise dominating microbial species, and hence increase in the diversity might be observed (Oono et al., 2020). In contrast, a high microbial abundance in roots could be attributed to the antimicrobial activity in the roots of *Arnebia* (Gupta et al., 2013), which might inhibit colonization by sensitive microbial communities, therefore reducing the overall diversity. Nonetheless, our data provided evidence of microbial richness based on the cultivability of these microorganisms. Further diversity analyses by enhancing the sample size and following culture-independent approach would throw more light on microbial community composition in different plant compartments.

### Functional Characterization of Endophytes

**FIGURE 8:**
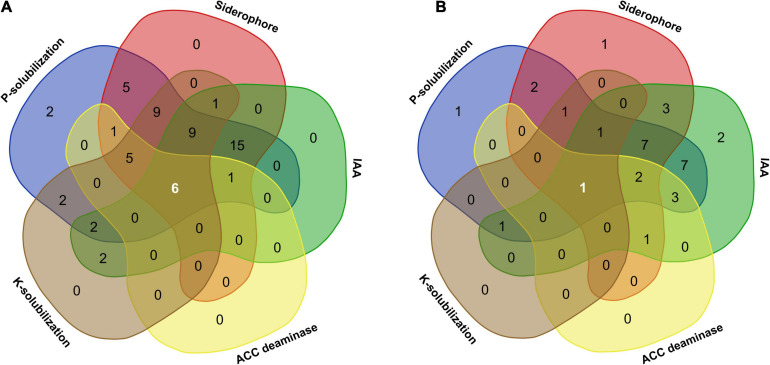
Venn diagram illustrating the number of microbial communities associated with *A. euchroma* possessing unique and overlapping PGP traits. **(A)** Bacterial endophytes with common and unique PGP traits. Among 60 bacterial endophytes, 6 bacteria displayed all PGP characters, while 15 isolates showed 3 PGP characters (P-solubilization, siderophore, and IAA production). **(B)** Fungal endophytes with common and unique PGP traits. Out of 33 fungal endophytes, only one isolate shared all PGP traits, while seven isolates showed three PGP characters (P-solubilization, siderophore, and IAA production).

Plant growth promotion by microbial endophytes inhabiting different plant tissues facilitates nutrient exchange (Hassan, 2017). In the present study, bacterial as well as fungal isolates showed different PGP activities, namely, ACCD, IAA, and siderophore production and solubilization of TCP and K. Of all the microbial endophytes, six bacteria (*Pseudomonas grimontii* ARB5, *Serratia plymuthica* ARB15, *P. frederiksbergensis* ARB19, *P. frederiksbergensis* ARB20, *P. grimontii* ARB36, and *P. grimontii* ARB38) (Figure 8A) and one fungal isolate (*Penicillium* sp. ARF9) (Figure 8B) possessed all the five PGP characteristics.

Microbial solubilization of inorganic phosphate is an important character mediating P uptake to the plants. In this study, P-solubilization by endophytic bacteria and fungi was considerably higher which ranged from 49.57 to 718.55 μg/ml and from 6.96 to 304.06 μg/ml, respectively. Further, a negative correlation between pH and soluble phosphorus (Figures 5C,E) clearly suggested the release of various organic acids produced in the course of P-solubilization by microbes (Adhikari et al., 2021). The abilities of microbial endophytes particularly those associated with root tissues of *A. euchroma*, which are anyway believed to be a specialized pool of rhizosphere microbes (Afzal et al., 2019), to solubilize higher P might be attributed to the low P availability in nutrient-depleted cold desert soils. Due to the alkaline pH and high calcium content of soils in cold deserts (Acharya et al., 2012), most of the soil P forms insoluble calcium phosphates, which are unavailable to plants. Evidence of the occurrence of P-solubilizing abilities in endophytes including bacteria and fungi is well documented (Ye et al., 2019; Varga et al., 2020). For instance, various PGP endophytic *Pseudomonas* spp. can solubilize moderate to high P (∼400–1,300 mg/L) (Oteino et al., 2015). In addition to P, microbial endophytes of *A. euchroma* also showed K-solubilization abilities; however, occurrence of these microbes was lower as compared to P solubilizers. A reduction in the pH of the surrounding environment through organic acid secretion by these microbes might account for the release of K ion from its mineral salts (Kour et al., 2020). The K-solubilizing efficiency of endophytic bacteria and fungi from a medicinal plant *Glycyrrhiza uralensis* was also reported (Li et al., 2018).

Hormones play a vital role in establishing a link between environment and plant response or phenotype (Dubois et al., 2018). Therefore, contribution of microbial endophytes in plant hormone signaling can mediate several benefits to the host plants, especially under environmental stress. A total of 20 endophytes that included 13 bacteria and 7 fungi displayed ACCD production in the present study (Figure 4). Endophytic bacteria belonging to *Pseudomonas* were the major producer of ACCD. Surprisingly, this enzyme has no known function in microbes (Glick et al., 1999) and it is solely involved in mediating the breakdown of stress-induced ACC into ammonia and α-ketobutyrate, therefore lowering the ethylene level in plants. Owing to the extreme cold and arid conditions in the natural habitat of *A. euchroma*, ACCD production by these endophytes can be attributed to a mutualistic partnership between microbes and plants to reduce cold and drought stress. The roles of ACCD-producing microbes were investigated in improving plant growth under abiotic stress conditions (Tavares et al., 2018; Zarei et al., 2020). For instance, an enhanced tolerance on plantlets of *Phaseolus vulgaris* to freezing conditions between −16 and −2°C was observed in response to inoculation by ACCD-producing endophytes (Tiryaki et al., 2019).

Production of phytohormone IAA was another interesting trait observed in the endophytic isolates of *A. euchroma*. As expected, the ability to produce a higher amount of IAA was more in root-associated endophytes as compared to leaf-associated endophytes (Figure 6). IAA as a signal molecule plays a key role in plant–microbe interactions (Hilbert et al., 2012) and under stress conditions (Aslam et al., 2020). Therefore, the occurrence of different IAA producers as endophytes in *A. euchroma* plant tissues may account for mediating plant signaling/interactions and stimulating the growth of plants under cold environmental conditions. Contributions of IAA production by different microbial endophytes have been investigated in several plant growth promotion studies (Chen et al., 2017; Turbat et al., 2020).

Next to phytohormones, endophytic isolates of *A. euchroma* also produced variable amount of siderophore under iron-deficient *in vitro* conditions (Figure 7). A possible explanation of the prevalence of siderophore production abilities in these endophytes is the iron-limiting conditions in the cold desert soils (Acharya et al., 2012). Therefore, these microbial endophytes may provide essential iron to the plant for performing various cell functions. In addition to iron, siderophores also have variable affinities toward other mineral elements such as Co, Mn, Mo, and Ni (Goswami et al., 2016), which provide endophytes with a competitive advantage over non-producers/pathogens to colonize the same ecological niche such as plant endosphere or rhizosphere. Moreover, siderophores can also trigger plant immunity under different stress conditions through induced systemic resistance in plant tissues including roots and leaves (Aznar and Dellagi, 2015).

Besides nutrient uptake, some of the endophytes isolated in the present study were earlier reported to confer stress tolerance in different plants. For example, a strain of *Pseudomonas frederiksbergensis* OS261 isolated from *Solanum lycopersicum* protects cells against chilling as well as salt stress (Subramanian et al., 2016; Chatterjee et al., 2017). Similarly, bacterial inoculation with *Brevibacterium frigoritolerans* reduced frost injury in *Phaseolus vulgaris* L. (Tiryaki et al., 2019). Therefore, the present study provided new opportunities to explore the abilities of these endophytes in improving abiotic stress tolerance in plants including agriculture crops.

## Conclusion

The present investigation is the first report on the diversity and composition analyses of endophytic microbes including bacteria, fungi, and yeasts associated with the root and leaf tissues of an endangered medicinal plant *Arnebia euchroma* (Royle) Johnston that experiences multiple extreme conditions in its natural habitat. This study concludes that, despite extremities in the habitat of *A. euchroma*, this plant species harbors a rich reservoir of microbial communities symbiotically residing inside different plant tissues. Leaf tissue of *A. euchroma* supported a more diverse array of cultivable endophytes as compared to root tissues. Overall, *Pseudomonas* and *Penicillium* were the most commonly observed bacterial and fungal genera, respectively. Various endophytic yeasts of Ascomycota and Basidiomycota phylum were also observed in leaf and root tissues. These endophytic microbes represent only a fraction of the microbial communities that could be cultivated while a large part (uncultivable endophytes) still remain unexplored due to their inability to culture and require new advancements. All the endophytes variably possessed one or more important PGP traits, viz., ACCD, IAA, and siderophore production and P and K solubilization. The presence of at least one plant-beneficial activity in all the isolated endophytes undoubtedly indicated the existence of a mutualistic relationship between *A. euchroma* plant and associated endophytes, supporting each other under extreme cold and arid environmental conditions. The present study opened new avenues for exploration of *A. euchroma*-associated endophytes for the amelioration of stress tolerance of the host plant. A more in-depth study is required to understand the host plant–endophyte–environment interactions and to develop specific microbial strategies for providing stress tolerance in plants.

## Data Availability Statement

The datasets presented in this study can be found in online repositories. The names of the repository/repositories and accession number(s) can be found in the article/Supplementary Material.

## Ethics Statement

This study including sample collection was conducted according to India’s Biological Diversity Act 2002 which permits the use of biological resources to bonafide Indians for scientific research purpose (Venkataraman, 2009).

## Author Contributions

RJ conceived and designed the experiments. RJ and PB performed the experiments, analyzed the data, and drafted the manuscript. SP and SK supervised and edited the manuscript. SK conceptualized the idea, generated the resources, and finalized the manuscript. All authors contributed to the manuscript revision and read and approved the submitted version.

## Conflict of Interest

The authors declare that the research was conducted in the absence of any commercial or financial relationships that could be construed as a potential conflict of interest.

## Footnotes

1 https://www.ezbiocloud.net/

2 http://www.ncbi.nlm.nih.gov

3 https://itol.embl.de/

4 http://jvenn.toulouse.inra.fr/app/index.html

## Supplementary Material

Supplementary Figure 1 Number of different bacterial **(A)** and fungal **(B)** endophytic genera isolated from leaf and root tissues of *Arnebia euchroma*.

Supplementary Figure 2 Different alpha diversity indices as calculated using PAST software after percentile type bootstrapping. Error bars indicates lower and upper values obtained after bootstrapping (*N* = 9,999). **(A)** Simpson’s index, **(B)** Shannon’s diversity index, **(C)** evenness, and **(D)** species richness of bacteria, fungi, and all microbial communities in different plant compartments and whole plant.

Supplementary Figure 3 Variability in the plant growth promoting traits of bacterial and fungal endophytes in leaf and root tissues. Range of P-solubilization by bacterial **(A)** and fungal **(B)** endophytes. Range of IAA production by bacterial **(C)** and fungal **(D)** endophytes. Range of siderophore production by bacterial **(E)** and fungal **(F)** endophytes.

Supplementary Table 1 Identification of bacterial endophytes isolated from *A. euchroma* and their nucleotide accession numbers.

Supplementary Table 2 Identification of fungal endophytes isolated from *A. euchroma* and their nucleotide accession numbers.

Supplementary Table 3 PGP traits of bacterial endophytes screened using plate-based assays.

Supplementary Table 4 PGP traits of fungal endophytes screened using plate-based assays.
