# Supplementary figures and images for: Arnebia euchroma, a Plant Species of Cold Desert in the Himalayas, Harbors Beneficial Cultivable Endophytes in Roots and Leaves
Source: Front Microbiol. 2021 Jul 16;12:696667. doi: 10.3389/fmicb.2021.696667 (PMC8322769; doi:10.3389/fmicb.2021.696667)

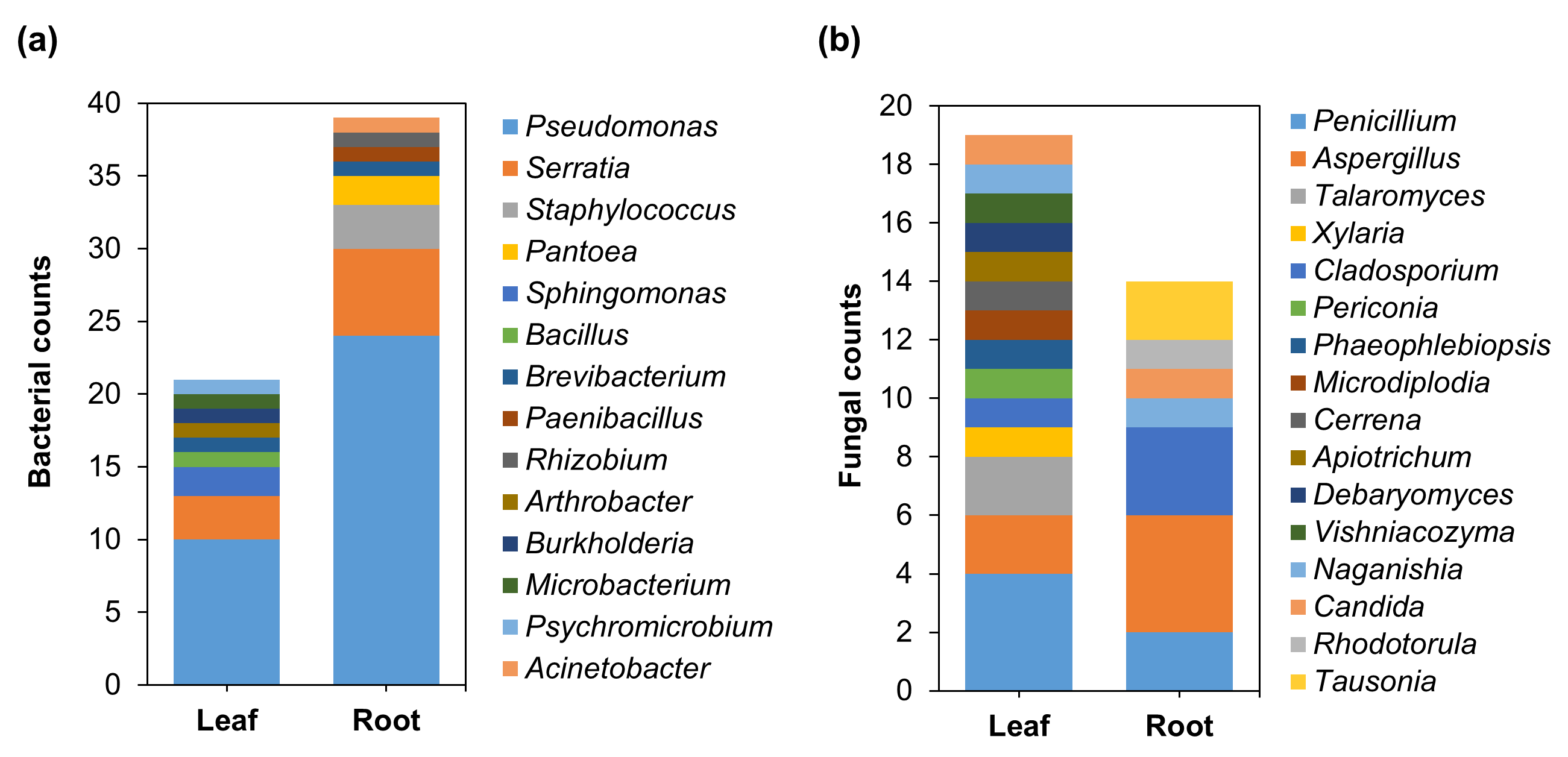

Supplement: Supplementary Figure 1 — Number of different bacterial (A) and fungal (B) endophytic genera isolated from leaf and root tissues of Arnebia euchroma. [file Image_1.TIF]

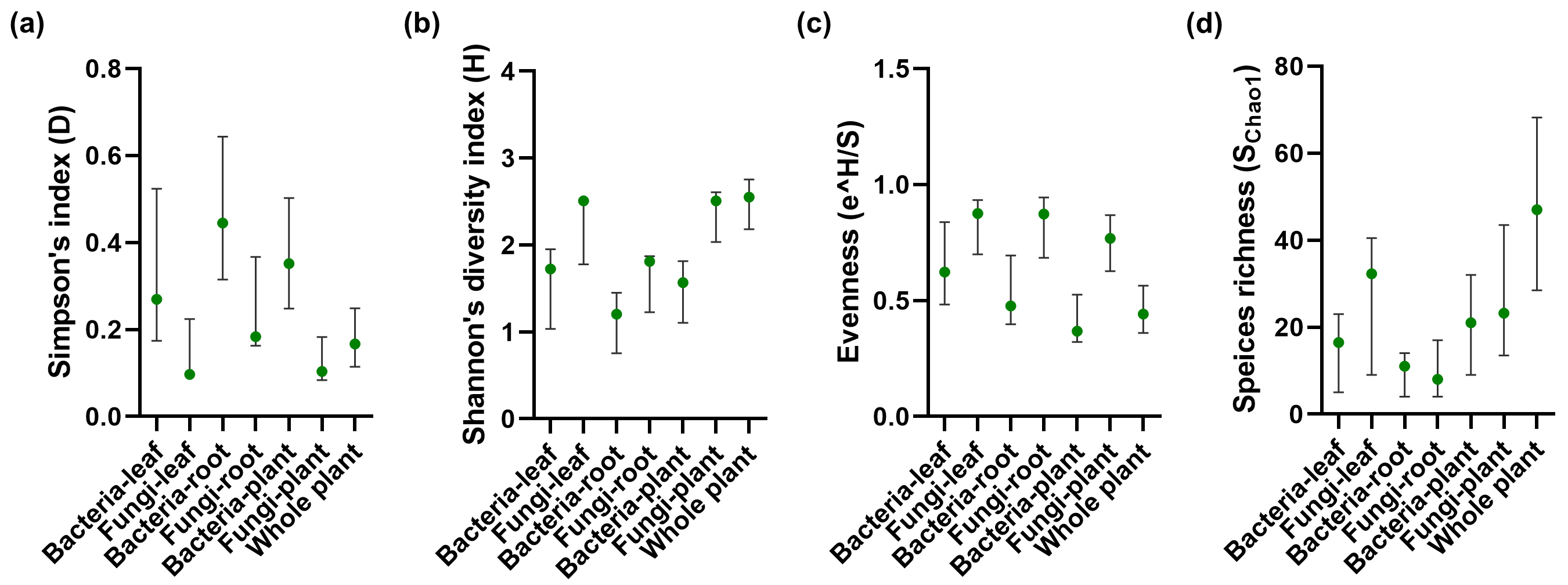

Supplement: Supplementary Figure 2 — Different alpha diversity indices as calculated using PAST software after percentile type bootstrapping. Error bars indicates lower and upper values obtained after bootstrapping (N = 9,999). (A) Simpson’s index, (B) Shannon’s diversity index, (C) evenness, and (D) species richness of bacteria, fungi, and all microbial communities in different plant compartments and whole plant. [file Image_2.TIF]

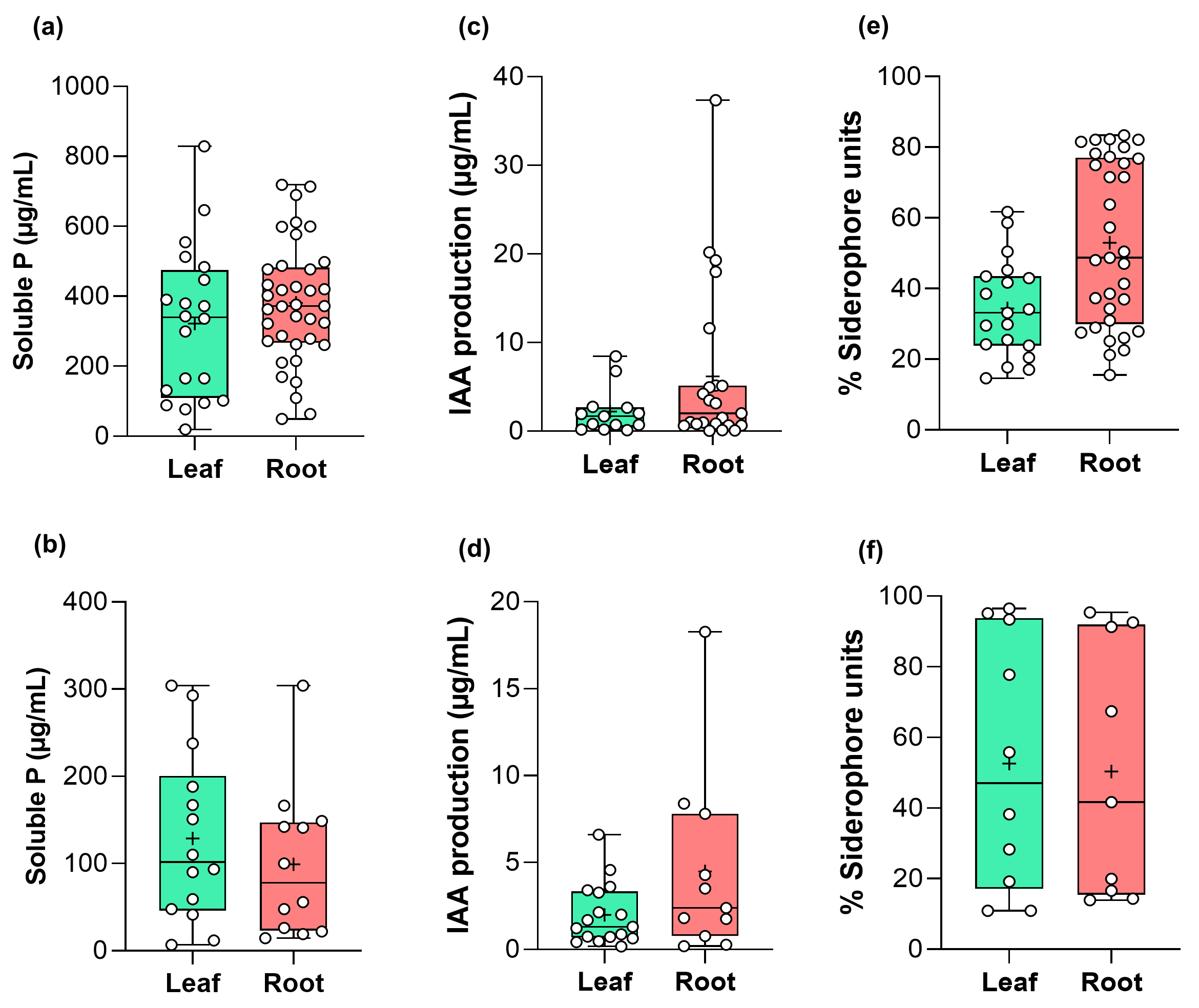

Supplement: Supplementary Figure 3 — Variability in the plant growth promoting traits of bacterial and fungal endophytes in leaf and root tissues. Range of P-solubilization by bacterial (A) and fungal (B) endophytes. Range of IAA production by bacterial (C) and fungal (D) endophytes. Range of siderophore production by bacterial (E) and fungal (F) endophytes. [file Image_3.TIF]
